# Biovera-Epi: A new database on species diversity, community composition and leaf functional traits of vascular epiphytes along gradients of elevation and forest-use intensity in Mexico

**DOI:** 10.3897/BDJ.9.e71974

**Published:** 2021-10-07

**Authors:** Valeria Guzmán-Jacob, Patrick Weigelt, Dylan Craven, Gerhard Zotz, Thorsten Krömer, Holger Kreft

**Affiliations:** 1 Biodiversity, Macroecology and Biogeography, University of Göttingen, Göttingen, Germany Biodiversity, Macroecology and Biogeography, University of Göttingen Göttingen Germany; 2 Universidad Mayor, Santiago, Chile Universidad Mayor Santiago Chile; 3 Universität Oldenburg, Oldenburg, Germany Universität Oldenburg Oldenburg Germany; 4 Centro de Investigaciones Tropicales, Universidad Veracruzana., Xalapa, Veracruz, Mexico Centro de Investigaciones Tropicales, Universidad Veracruzana. Xalapa, Veracruz Mexico

**Keywords:** elevational gradient, vascular epiphytes, functional traits, forest-use intensity, carbon isotope ratio, nitrogen isotope ratio.

## Abstract

**Background:**

This data paper describes a new, comprehensive database (BIOVERA-Epi) on species distributions and leaf functional traits of vascular epiphytes, a poorly studied plant group, along gradients of elevation and forest-use intensity in the central part of Veracruz State, Mexico. The distribution data include frequencies of 271 vascular epiphyte species belonging to 92 genera and 23 families across 120 20 m × 20 m forest plots at eight study sites along an elevational gradient from sea level to 3500 m a.s.l. In addition, BIOVERA-Epi provides information on 1595 measurements of nine morphological and chemical leaf traits from 474 individuals and 102 species. For morphological leaf traits, we provide data on each sampled leaf. For chemical leaf traits, we provide data at the species level per site and land-use type. We also provide complementary information for each of the sampled plots and host trees. BIOVERA-Epi contributes to an emerging body of synthetic epiphytes studies combining functional traits and community composition.

**New information:**

BIOVERA-Epi includes data on species frequency and leaf traits from 120 forest plots distributed along an elevational gradient, including six different forest types and three levels of forest-use intensity. It will expand the breadth of studies on epiphyte diversity, conservation and functional plant ecology in the Neotropics and will contribute to future synthetic studies on the ecology and diversity of tropical epiphyte assemblages.

## Introduction

Elevational gradients provide a wide range of opportunities for studying the effects of different ecological and evolutionary factors on biodiversity patterns. Steep elevational gradients in temperature, precipitation and other climatic variables usually play a fundamental role in shaping plant diversity (McCain and Grytnes 2010, Peters et al. 2019) and also contribute to linkages between plant traits and environmental conditions (Bruelheide et al. 2018, Keddy 1992). They are also used as proxies for understanding diversity patterns across latitudinal gradients (McCain and Grytnes 2010), while controlling for species pools and biogeographic history (Ricklefs 2004). Nevertheless, anthropogenic forest disturbance may modify climatic conditions at local and regional scales which, in turn, may affect the response of species, causing upward shifts in the treeline (Cazzolla Gatti et al. 2019), shifting the distribution of plants and animals (McCain et al. 2016) and might be especially threatening for canopy-dwelling life forms, such as vascular epiphytes that are sensitive to changes in air humidity and temperature (Larrea and Werner 2010, Werner and Gradstein 2009, Zotz and Bader 2009). Furthermore, while a growing number of studies shows that climate change affects a wide range of species and ecosystems (Peters et al. 2019, Root et al. 2003, Trisos et al. 2020, Walther et al. 2002), few studies focus on vascular epiphytes and their composition, diversity and functional traits, especially comparing different levels of forest-use intensity.

Functional traits are measurable characteristics of individual plants impacting their growth, reproduction and survival (Violle et al. 2007) and reflect how species interact with their environment (Vesk 2013). Functional traits are widely used to elucidate mechanisms that underpin many ecological processes along vertical and horizontal environmental gradients (e.g. Petter et al. 2015, Bruelheide et al. 2018), but also evolutionary patterns associated with variation in plant form and function, such as geographic distributions of woody and non-woody species (Díaz et al. 2015). Despite recent progress (e.g. Agudelo et al. 2019, Petter et al. 2015), studies in the field of functional traits of vascular epiphytes are rare, suggesting that our knowledge of the factors that determine the distribution of vascular epiphytes along environmental gradients is similarly limited.

Mexico is a country with high floristic diversity and endemism. Almost 50% of its 23,114 native species of vascular plants are endemic. However, Mexico has lost approximately half of its forest cover in the past 50 years (Barsimantov and Kendall 2012). Furthemore, it has been estimated that about 7.8% of the Mexican vascular flora are epiphytes, 750 of which (569 angiosperms and 181 pteridophytes) are native to Veracruz (Krömer et al. 2020). Vascular epiphytes usually reach their highest diversity in humid tropical forests at mid-elevations (Guzmán‐Jacob et al. 2019,Küper et al. 2004, Körmer et al. 2005, Cardelús et al. 2006), (Fig. 3). Moreover, they contribute significantly to ecosystem functioning through biotic interactions and by providing microhabitats for other organisms (Nadkarni 1984, Veneklaas et al. 1990, Zotz 2016).

Even when epiphytes represent about 9% of all vascular plant species (Zotz 2016), they are strongly under-represented in global traits datasets. With this study, we aim at contributing to the percentage of epiphyte species represented in global datasets. We believe that the assemblage of local information in global databases covering species occurrences and functional traits can help to validate ecological theories at larger scales. In particular, the inclusion of an increasing number of studies on functional ecology can foster new frameworks and theories to better understand how biodiversity responds to an increasingly fragmented natural world. Here, we describe a new database on species distributions and leaf functional traits of vascular epiphytes along gradients of elevation and levels of forest-use intensity.

## General description

### Purpose

BIOVERA-Epi includes plot data from an elevational gradient located in the central part of the State of Veracruz, Mexico. Specifically, it contains two distinct, but related datasets: the first dataset includes distribution and plot level frequency information (frequency.subplot) for 271 vascular epiphyte species, sampled in 120 20 m × 20 m plots along the elevational gradient, ranging from 0 to 3500 m a.s.l. The second dataset includes measurements of nine morphological and chemical leaf traits for 102 species, 474 individuals and a total of 1595 leaves, which were sampled in 45 plots at three sites along the same elevational gradient. The leaf traits studied were: leaf area, leaf density, specific leaf area (SLA), leaf dry matter content (LDMC), leaf nitrogen content, leaf phosphorus content, leaf carbon content, nitrogen isotope ratio (d^15^N) and carbon isotope ratio (d^13^C). For each plot, we also provide geographical coordinates, forest-use intensity (old-growth, degraded, secondary) and elevation. For the surveyed host trees, we report diameter at breast height (DBH), total height (H) and species identity (see Data collection).

**Conclusion**: The species distribution dataset shows the value of old-growth forest for epiphyte diversity, but also show that degraded and secondary forest, depending on the elevation, may maintain a high species diversity and thus play an important role in conservation planning. Across our 120 study plots, Orchidaceae was the family with more species within the Angiosperms and Polypodiaceae within the Pteridophytes (Fig. 4). Furthermore, the leaf trait dataset shows the leaf trait variation among families, where some families show larger variation than others for both morphological and chemical traits (Figs 5, 6).

## Sampling methods

### Sampling description

**Sampling design**

**Figure 1.**
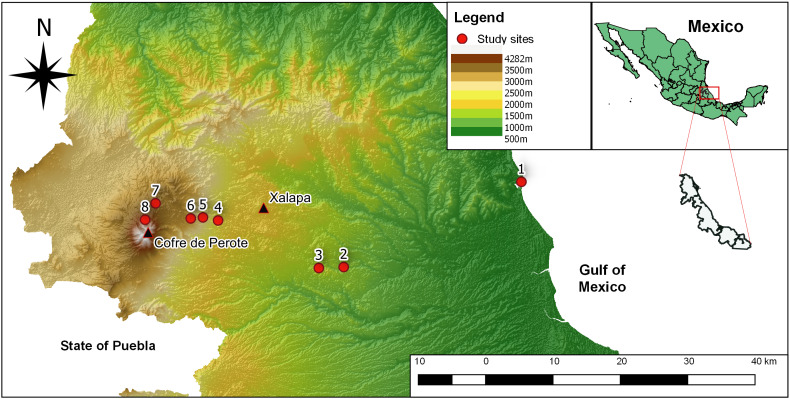
Map of the study sites along the eastern slopes of the Cofre de Perote mountain in the State of Veracruz, Mexico. Red dots indicate the location of the eight study sites. Black triangles indicate the summit of the Cofre de Perote mountain and the City of Xalapa as reference points.

The elevational gradient spanned from sea level to 3500 m on the eastern slopes of Cofre de Perote, a 4282 m extinct volcano located in the central part of Veracruz State, Mexico (Fig. 1). In this region, the Trans-Mexican volcanic belt and the Sierra Madre Oriental converge, creating complex geological conditions and combining floristic elements from the Nearctic and Neotropics. The climate in the study region ranges from dry and hot in the lowlands (mean annual temperature (MAT): 25°C; mean annual precipitation (MAP): 1222 mm yr^-1^) to humid and temperate at mid-elevations (MAT: 13-19°C; MAP: 2952-1435 mm yr^-1^) and dry and cold at high elevations (MAT: 9°C; MAP: 708 mm yr^-1^; data according to the National Meteorological Service of Mexico 1951-2010). Along the elevational gradient, six main vegetation types are commonly found (Carvajal-Hernández and Krömer 2015): (1) semi-humid deciduous forest at 0-700 m, (2) tropical oak forest at 700-1300 m, (3) humid montane forest at 1300-2400 m, (4) pine-oak forest at 2400-2800 m, (5) pine forest at 2800-3500 m and (6) fir forest at 3500-3600 m.

We investigated three levels of forest-use intensity (FUI) that could be consistently found along the entire gradient (following Gómez-Díaz et al. 2017): (1) old-growth forests (OG) encompass mature forests with no or only few signs of logging and other human impacts and are classified as the lowest FUI; (2) degraded forests (DF) are forests with clear signs of past logging, sometimes with ongoing cattle grazing, removal of understorey and/or harvesting of non-timber forest products and are classified as intermediate FUI; and (3) secondary forests (SF) represent forests at an intermediate successional stage 15-25 years after abandonment (based on interviews with local landowners), often with signs of continued human impacts, such as the removal of understorey vegetation, non-timber forest products or partial tree cutting and occasional cattle grazing and are classified as high FUI.

**Data collection: species distribution**

**Figure 2.**
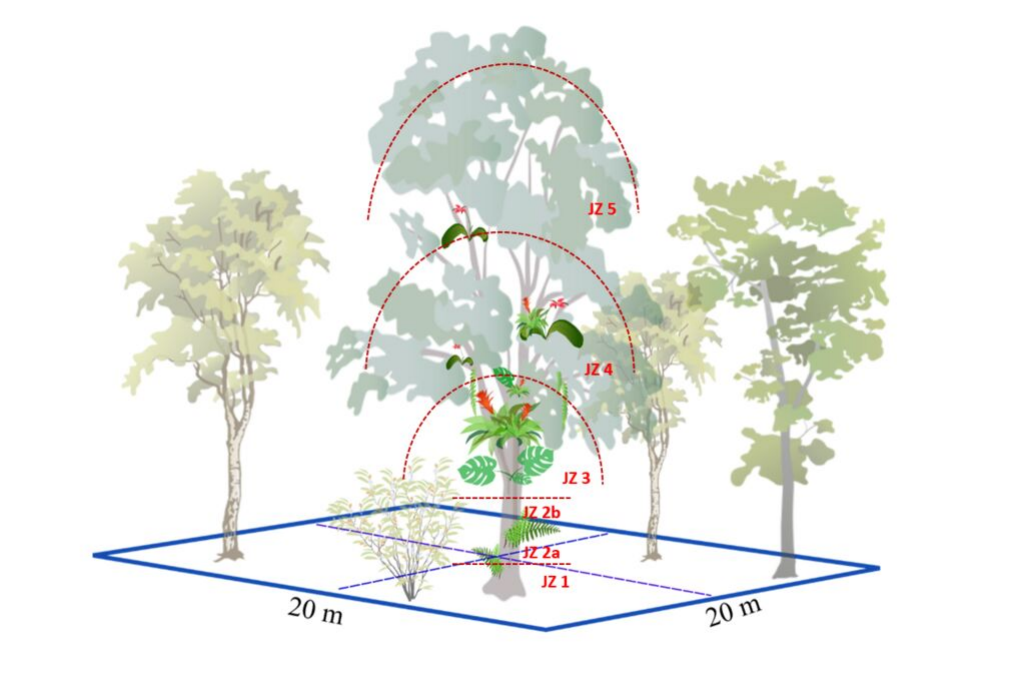
Design of a 20 × 20 m plot for sampling vascular epiphytes. The four subplots are indicated by dashed blue lines. The central tree shows the five Johansson zones indicated with red lines, base of the trunk (JZ 1), lower trunk (JZ 2a), upper trunk (JZ 2b), inner canopy (JZ 3), mid-canopy (JZ 4) and outer canopy (JZ 5). We used the adapted version of the system, where the trunk is divided into two separate zones (ter Steege and Cornelissen 1989).

**Figure 3.**
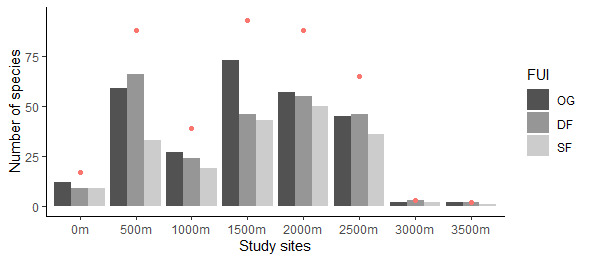
Total species number per elevation and forest-use intensity. Number of species of vascular epiphytes recorded at the different levels of forest-use intensity (FUI: OG; old-growth forest, DF; degraded forest and SF; secondary forest) at each of the study sites (0 m, 500 m, 1000 m, 1500 m, 2000 m, 2500 m, 3000 m and 3500 m). At each elevational site, five plots were sampled per FUI. Red points indicate the total number of species per study site.

**Figure 4.**
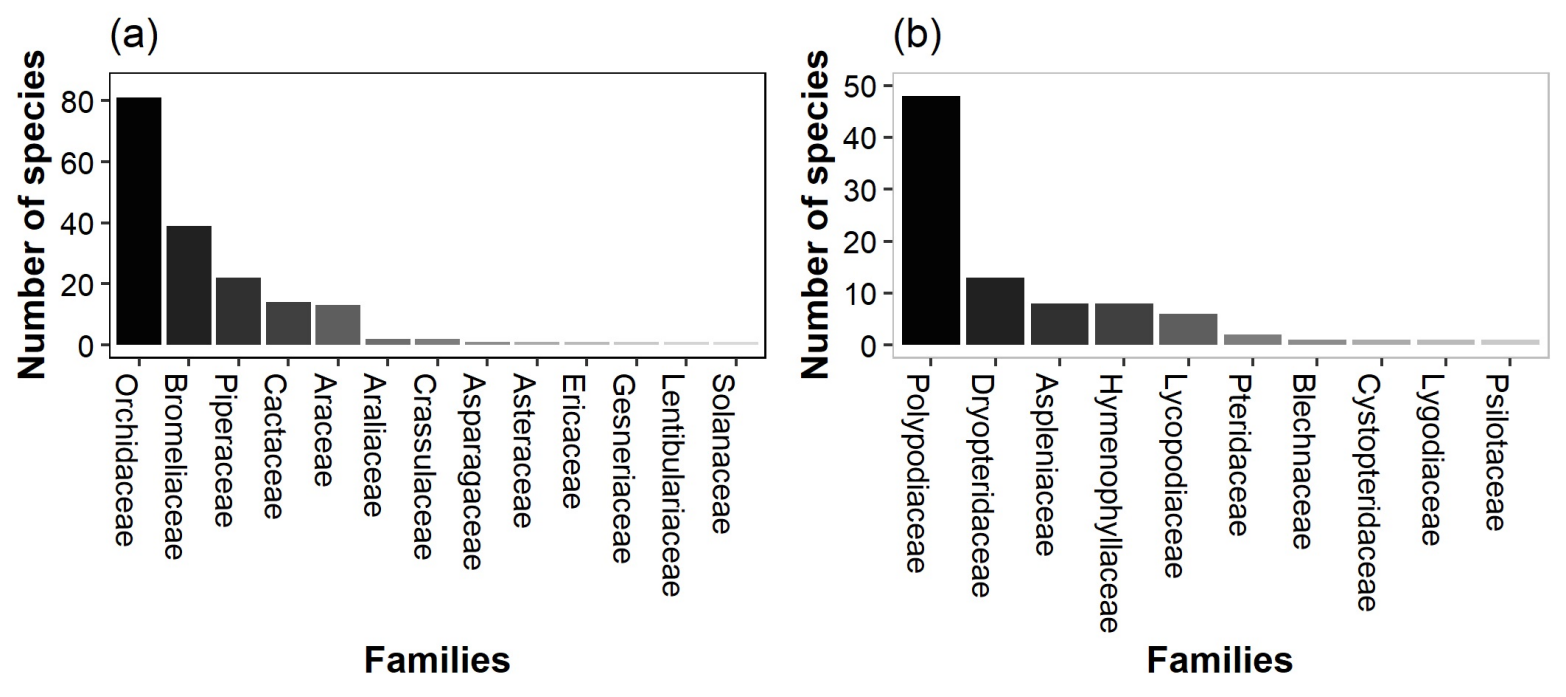
Total number of species per family recorded in the 120 plots: a) Angiosperms, (b) Pteridophytes. Note the different scales of the y-axes.

We selected eight study sites, each separated by ca. 500 m in altitude along the elevational gradient, representing the following elevational ranges: 0-45 m, 610-675 m, 980-1050 m, 1470-1700 m, 2020-2200 m, 2470-2600 m, 3070-3160 m and 3480-3545 m. At each study site, we surveyed vascular epiphytes in five non-permanent 20 m × 20 m plots for each of the three FUI levels, respectively, yielding a total of 120 plots (Suppl. material 1). We used a Garmin® GPSMAP 60Cx device (Garmin International, Inc. Kansas, USA) to record geographical coordinates and elevation for all plots. Vascular epiphytes were surveyed between July 2014 and May 2015 following the sampling protocol of Gradstein et al. 2003. First, ground-based surveys were conducted in four 10 m × 10 m subplots nested within each plot, to represent epiphyte assemblages in the forest understorey up to a height of ~ 6 m (Krömer et al. 2006, Krömer and Gradstein 2016) using collecting poles and binoculars (Flores-Palacios and Garca-Franco 2001). We selected one mature host tree per plot, based on size, vigour and crown structure for safe canopy access. We climbed from the base to the outer portion of the tree crown using the single-rope climbing technique (Perry 1978) and recorded the presence of vascular epiphyte species in each of the five vertical tree zones according to Johansson (1974), (Fig. 2). Johansson zones are a frequently-used scheme to record and describe the spatial distribution of vascular epiphytes within tree trunks and canopies (Gradstein et al. 2003, Sanger and Kirkpatrick 2016). We recorded diameter at breast height (DBH) and total height for each climbed tree. We recorded the frequency of each species as the sum of incidences in the four nested subplots (frequency.subplot, maximum frequency per plot = 4) (Suppl. material 2, Figs 3, 4). We also recorded the frequency of each species as the sum of incidences in the five Johansson zones of the central host tree (frequency.J.zones, maximum frequency = 5).

**Data collection leaf trait dataset**

In a separate sampling campaign from June to September 2016, leaf trait sampling took place at three of our studied elevational sites (0, 500 and 1500 m a.s.l.). In this field campaign, we aimed to resample as many vascular epiphyte species from the first survey as possible. At each elevation, epiphytes were sampled up to a height of 20 m on one or more trees using the single-rope climbing technique. Epiphytes below 6 m were sampled from the ground using a collecting pole. Functional traits were collected for all vascular epiphyte species classified as holoepiphytes (epiphytes in the strict sense, i.e. living their whole life cycle as epiphytes). In this dataset, we excluded nomadic vines because of their contact with the ground (Zotz 2013). Additionally, we excluded species of the family Cactaceae from trait measurements because stems are their main photosynthetic organs. This dataset differs in the sampling resolution between morphological and chemical traits; morphological traits include leaf measurements per individual at each study site and chemical traits include one measurement (from pooled samples) per species from each study site.

**Leaf trait measurements**

**Figure 5.**
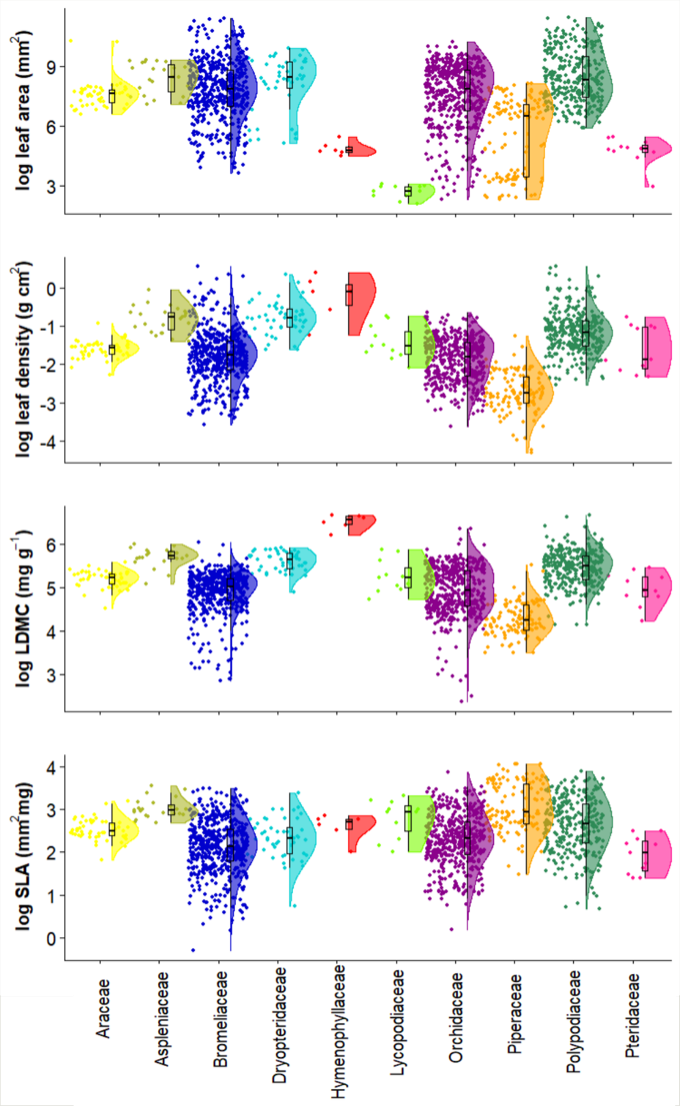
Morphological leaf traits per family. Distribution of trait measurements across the 102 species and 10 families at 500, 1500 and 2500 m. Each point represents a leaf measurement (n = 1595).

**Figure 6.**
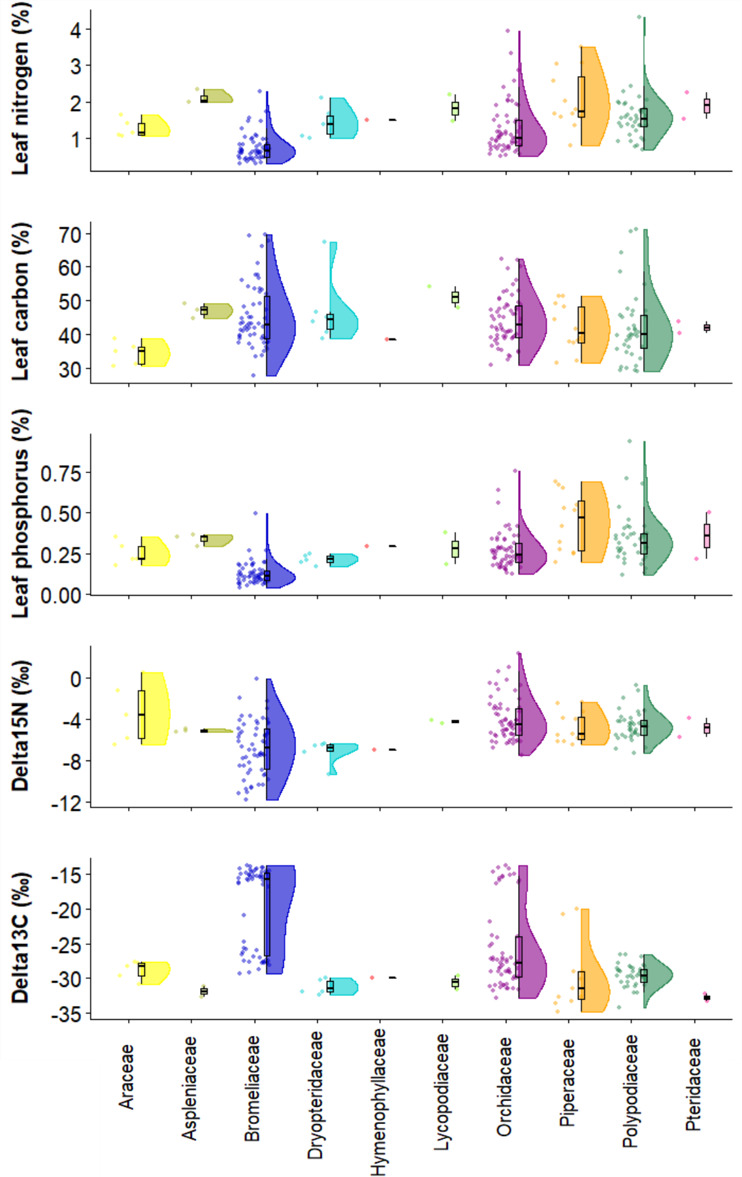
Chemical leaf traits per family. Distribution of trait measurements across the 102 species and 10 families at 500, 1500 and 2500 m. Each point represents a species measurement (n = 189).

We collected between one and three leaves per adult individual from three individuals to obtain, if possible, a maximum of 10 leaves per species. We sampled fully expanded leaves without visible signs of herbivory or disease. Collected leaves were rehydrated in a sealed plastic bag and kept cool in a refrigerator at 7°C for a minimum of 8 hours before taking measurements. Leaf area was measured with a portable laser area meter (CI-202, CID Bio Science Inc. U.S.A.). Leaf thickness was measured with an electronic calliper (precision: 0.05 mm). Leaves were weighed to obtain fresh weight (balance: A and D GR-202; A and D Company, Tokyo, Japan; precision: 0.1 mg), then oven-dried at 70°C for 48 h or until obtaining a constant dry weight and reweighed to obtain dry weight. For each leaf, we determined the following morphological traits following Prez-Harguindeguy et al. 2013 and [Bibr B7159030][Bibr B6772965], Kitajima and Poorter 2010: i) leaf area (LA = mm) , ii) specific leaf area (SLA = leaf area/dry weight; mm/mg) , iii) leaf density (LD = SLA/leaf thickness; g/cm) and iv) leaf dry matter content (LDMC = dry weight/fresh weight; g/g) (Suppl. material 3, Fig. 5). We measured the following leaf chemical traits: i) leaf nitrogen content (leaf nitrogen; %), ii) leaf carbon content (leaf carbon; %), iii) leaf phosphorus content (leaf phosphorus; %), iv) nitrogen isotope ratio (d^15^N; ‰), and v) carbon isotope ratio (d^13^C; ‰) (Suppl. material 4, Fig. 6). Dried leaf samples were ground and homogenised using a ball mill. To quantify leaf nitrogen content, leaf carbon content, d^15^N and d^13^C, we used an elemental analyser-isotope ratio mass spectrometer (Carlo Erba 1110 EA coupled via a Conflo III to a Delta ^PLUS^; Thermo Electron, Bremen, Germany). We used an internal standard, which is a solution of proline and sucrose with a C:N ratio of 8.8, d^15^N of 0.16 (+/i 0.15) and d^13^C of -10.20 (+/-0.13). We tested standards every ten samples, after which the IRMS was recalibrated using five certified isotope standards, i.e. IAEA-600, IAEA-N-1, IAEA-N2 and USGS-25. Atmospheric air (AIR) was used for d^15^N and the Vienna Pee Dee Belemnite (VPDB) for d^13^C as standards.

d^13^C (‰) = [(^13^C/^12^C sample)/ (^13^C/^12^C standard)-1] × 1000

d^15^N (‰) = [(^15^N/^14^N sample)/ (^15^N/^14^N standard)-1] × 1000

To determine leaf phosphorus, 5 mg of the sample were digested in 200 μl concentrated nitric acid (HNO_3_) and 30 μl 30% hydrogen peroxide (H_2_O_2_) (Huang and Schulte 1985). Leaf phosphorus concentrations were determined colourimetrically (Murphy and Riley 1962). After digestion, 770 μl distilled water was added and the absorption by the molybdenum-phosphorus complex was measured at 710 nm using a UV-VIS spectrophotometer (Specord 50, Analytik Jena, Jena, Germany). Chemical analyses of samples were performed at the University of Oldenburg for phosphorus and at the University of Vienna, Department of Microbiology and Ecosystem Science for nitrogen, d^15^N and d^13^C.

**Species identification**

Vouchers from the first field campaign were collected, if possible, in triplicate for preservation as herbarium specimens. These specimens were identified using relevant literature (Croat and Acebey 2015, Espejo-Serna et al. 2005,Hietz and Hietz-Seifert 1994, Mickel and Smith 2004) and by comparison with specimens deposited at the National Herbarium (MEXU) and Universidad Nacional Autónoma de México in Mexico City and the herbarium of the Institute of Ecology (XAL) in Xalapa. Some taxa were sent to the following specialists for identification: Crassulaceae (Dr. Pablo Carrillo-Reyes, Universidad de Guadalajara), Cactaceae (Dr. Miguel Cházaro-Bazáñez, Universidad Veracruzana), Bromeliaceae and Orchidaceae (Dr. Adolfo Espejo-Serna and MSc. Ana Rosa López-Ferrari, Universidad Autónoma de México, Iztapalapa), Pteridophytes (Dr. Alan Smith, UC Berkeley, USA) and Peperomia (Guido Mathieu, Botanic Garden Meise, Belgium). Species not identified to species level were assigned to morphospecies, using the genus or family name followed by the registered elevation and a consecutive number (Suppl. material 5). The collection of species protected by Mexican Law was facilitated by a plant collection permit (NOM-059-SEMARNAT-2010) issued by the Secretaría de Medio Ambiente y Recursos Naturales (SEMARNAT SGPA/DGVS/2405/14). All scientific names follow The Plant List version 1.1 (2013).

## Geographic coverage

### Description

Data were collected at eight different sites distributed across an elevational gradient along the eastern slopes of Cofre de Perote mountain, Veracruz State, Mexico.

### Coordinates

19.51 Latitude and -96.15 Longitude Latitude; -96.38 Longitude and 19.59 Latitude Longitude.

## Taxonomic coverage

### Description

1) Epiphytes: The species distribution dataset covers 271 epiphyte species belonging to 92 genera and 23 families. The most species-rich families are Orchidaceae (82 species), Polypodiaceae (50), Bromeliaceae (41), Piperaceae (20), Cactaceae (14) and Araceae (12). A total of 72.2% of the sampled epiphyte individuals could be identified to species level, while another 26.1% were identified to genus level and 1.7% to family level. The trait dataset includes measurements for 1595 leaves from 474 individuals belonging to 102 species in 10 families. In total, most species were orchids (42.7%), followed by ferns (28.1%) and bromeliads (20.4%).

2) Phorophytes: The 120 climbed host trees belong to 32 tree species distributed in 25 genera and 21 families. Tree identification to the species level was possible in 53% of the cases, while another 44% were identified to genus level and 3% to family level.

## Usage licence

### Usage licence

Open Data Commons Attribution License

## Data resources

### Data package title

BIOVERA-Epi, a new database on species diversity, community composition and leaf functional traits of vascular epiphytes along an elevational gradient in Mexico

### Number of data sets

5

### Data set 1.

#### Data set name

Plot table

#### Number of columns

10

#### Description

Location of the 120 forest plots along the elevational gradient at the eastern slopes of Cofre de Perote mountain, Veracruz, Mexico (Suppl. material 1)

**Data set 1.** 

Column label	Column description
Plot_ID	ID of each plot
Vegetation	Vegetation type
FUI	Forest-use intensity
Site	Name of the study site
Elevation.precise	Metres above sea level
Latitude	Geographic coordinate
Longitud	Geographic coordinate
Tree.name	Scientific name of the central tree
DBH	Diameter at breast height in centimetres
Tree.height	Height of the tree in metres

### Data set 2.

#### Data set name

Distribution table

#### Number of columns

10

#### Description

Distribution data of 271 vascular epiphyte species at each plot along the elevational gradient and three levels of forest-use intensity (n = 5 plots per forest-use intensity within each elevation) (Suppl. material 2).

**Data set 2.** 

Column label	Column description
Plot_ID	ID of each plot
Sp.code	Code for each scientific species name
Frequency	The sum of incidences in the four nested subplots (maximum frequency per plot = 4)
JZone1	Johansson zone 1
JZone2a	Johansson zone 2a
JZone2b	Johansson zone 2b
JZone3	Johansson zone 3
JZone4	Johansson zone 4
JZone5	Johansson zone 5
Frequency.J.zones	The sum of incidences in the Johansson zones (maximum frequency = 5)

### Data set 3.

#### Data set name

Morphological leaf traits

#### Number of columns

9

#### Description

Single leaf trait measurements (leaf area, leaf density, specific leaf area and leaf dry matter content) per 474 individuals of 102 species and a total of 1595 leaves (Suppl. material 3).

**Data set 3.** 

Column label	Column description
Site	Name of the study site
FUI	Forest-use intensity
Sp.code	Code for each scientific species name
Ind.number	Number of the individual
Leaf.number	Number of the leaf
LA	Leaf area
LD	Leaf density
SLA	Specific leaf area
LDMC	Leaf dry matter content

### Data set 4.

#### Data set name

Chemical leaf traits

#### Number of columns

8

#### Description

Chemical leaf trait measurements (leaf nitrogen content, leaf phosphorus content, leaf carbon content, nitrogen isotope ratio and carbon isotope ratio) per 102 species (Suppl. material 4).

**Data set 4.** 

Column label	Column description
Site	Name of the study site
FUI	Forest-use intensity
Sp.code	Code for each scientific species name
Leaf nitrogen	Leaf nitrogen content
Leaf carbon	Leaf carbon content
Leaf.phosphorus	Leaf phosphorus content
Delta15N	Nitrogen isotope ratio
Delta13C	Carbon isotope ratio

### Data set 5.

#### Data set name

Species names

#### Number of columns

3

#### Description

Species scientific name and its corresponding family and species code (Suppl. material 5).

**Data set 5.** 

Column label	Column description
Species.code	Code for each scientific species name
Species.name	Scientific name of the species
Family	Family of the species

## Additional information

We provide the description of the content and structure of each supplementary material in Table 1, with the source of standardisation for each term used according to Darwin Core glossary and the Thesaurus of Plant Characteristics.

## Supplementary Material

D2194EA0-1FB0-50BF-B1ED-D244344FA8E3 10.3897/BDJ.9.e71974.suppl1 Supplementary material 1 Plot table Data type Plot information Brief description Location of the 120 forest plots along the elevational gradient at the eastern slopes of Cofre de Perote mountain, Veracruz, Mexico. File: oo_568751.csv https://binary.pensoft.net/file/568751 Valeria Guzmán-Jacob, Patrick Weigelt, Dylan Craven, Gerhard Zotz, Thorsten Krömer & Holger Kreft

4CDA3B59-1F13-51A0-91C3-8FD0A095FAEF 10.3897/BDJ.9.e71974.suppl2 Supplementary material 2 Distribution table Data type Distribution data Brief description Distribution data of 271 vascular epiphyte species at each plot along the elevational gradient and three levels of forest-use intensity (n = 5 plots per forest-use intensity within each elevation). File: oo_568752.csv https://binary.pensoft.net/file/568752 Valeria Guzmán-Jacob, Patrick Weigelt, Dylan Craven, Gerhard Zotz, Thorsten Krömer & Holger Kreft

7C2D7619-27DE-596B-A5D6-7710AF2398F5 10.3897/BDJ.9.e71974.suppl3 Supplementary material 3 Morphological leaf traits Data type Leaf traits Brief description Single leaf trait measurements (leaf area, leaf density, specific leaf area and leaf dry matter content) per 474 individuals of 102 species and a total of 1595 leaves. File: oo_568753.csv https://binary.pensoft.net/file/568753 Valeria Guzmán-Jacob, Patrick Weigelt, Dylan Craven, Gerhard Zotz, Thorsten Krömer & Holger Kreft

54417C18-852C-5898-91DE-D7B500A27638 10.3897/BDJ.9.e71974.suppl4 Supplementary material 4 Chemical leaf traits Data type Chemical leaf traits Brief description Chemical leaf trait measurements (leaf nitrogen content, leaf phosphorus content, leaf carbon content, nitrogen isotope ratio and carbon isotope ratio) per 102 species. File: oo_568754.csv https://binary.pensoft.net/file/568754 Valeria Guzmán-Jacob, Patrick Weigelt, Dylan Craven, Gerhard Zotz, Thorsten Krömer & Holger Kreft

F782E806-7A45-542A-95EC-BA0F83D64EB0 10.3897/BDJ.9.e71974.suppl5 Supplementary material 5 Species names Data type species list Brief description Species scientific name and its corresponding family and species code. File: oo_568755.csv https://binary.pensoft.net/file/568755 Valeria Guzmán-Jacob, Patrick Weigelt, Dylan Craven, Gerhard Zotz, Thorsten Krömer & Holger Kreft

## Figures and Tables

**Table 1.** Data documentation with information that describes the content and structure of each of the previous tables. The source of standardisation for each term used is provided in the *Standardized according to* column based on the Darwin Core glossary and the Thesaurus of Plant Characteristics (TOP). The name of the standardised term in the *Standardized Term* column. The term used in the present study in the *Term in this study* column. A definition is provided in the *Definition* column (following the Darwin Core, Thesaurus of Plant Characteristics or the given reference) and, if applicable, the unit of measurement in the *Unit* column.

**Standardised according to**	**Standardised Term**	**Term in this study**	**Definition**	**Unit**
Darwin Core	Family	Family	The full scientific name of the family in which the taxon is classified.	
Darwin Core	Habitat	Vegetation	A category or description of the habitat in which the Event occurred.	
Darwin Core	locationID	Plot_ID	An identifier for the set of location information (data associated with dcterms: Location). May be a global unique identifier or an identifier specific to the dataset.	
Darwin Core	Locality	Site	The specific description of the place. Less specific geographic information can be provided in other geographic terms (higherGeography, continent, country, stateProvince, county, municipality, waterBody, island, islandGroup). This term may contain information modified from the original to correct perceived errors or standardise the description.	
Darwin Core	organismID	Sp.code	An identifier for the Organism instance (as opposed to a particular digital record of the Organism). May be a globally unique identifier or an identifier specific to the dataset.	
Darwin Core	organismQuantityType	Frequency.subplot Frequency.J.zones	The type of quantification system used for the quantity of organisms.	
Darwin Core	scientificName	Species name / Tree name	The full scientific name, with authorship and date information, if known. When forming part of an Identification, this should be the name in lowest level taxonomic rank that can be determined. This term should not contain identification qualifications, which should instead be supplied in the IdentificationQualifier term. Note: we used a mixture of valid scientific names and informal names for plants not identified to the species level, therefore species names are not strictly Darwin Core-compliant.	
Darwin Core	verbatimElevation	Elevation	The original description of the elevation (altitude, usually above sea level) of the Location.	metres above sea level (m a.s.l.)
Darwin Core	DecimalLatitude	Latitude	The geographic latitude (in decimal degrees, using the spatial reference system given in geodeticDatum) of the geographic centre of a Location. Positive values are north of the Equator; negative values are south of it. Legal values lie between -90 and 90, inclusive.	
Darwin Core	DecimalLongitude	Longitude	The geographic longitude (in decimal degrees, using the spatial reference system given in geodeticDatum) of the geographic centre of a Location. Positive values are east of the Greenwich Meridian; negative values are west of it. Legal values lie between -180 and 180, inclusive.	
Functional Diversity thesaurus	Plant height trait	Height	the height (PATO:height) of a whole plant (PO:whole plant)	m
Functional Diversity thesaurus	Leaf density	Lamina density (LD)	leaf dry mass per leaf volume	g/cm^3^
Functional Diversity thesaurus	Leaf area	Leaf area (LA)	the area (PATO:area) of a leaf (PO:leaf) in the one sided projection	mm^2^
Functional Diversity thesaurus	Leaf dry matter content	Leaf dry matter content (LDMC)	the ratio of the dry mass of a leaf (TOP:leaf dry mass) to its water saturated fresh mass	g g^-1^
Functional Diversity thesaurus	Specific leaf area	Specific Leaf Area (SLA)	the ratio of the area of a leaf (TOP:leaf area) to its dry mass (TOP:leaf dry mass)	mm^2^ mg^-1^
Functional Diversity thesaurus	Leaf nitrogen content per leaf dry mass	Leaf nitrogen content	The ratio of the quantity of nitrogen of a leaf per unit dry mass.	%
Functional Diversity thesaurus	Leaf carbon content per leaf dry mass	Leaf carbon content	The ratio of the quantity of carbon of a leaf per unit dry mass.	%
Functional Diversity thesaurus	Leaf phosphorus content per leaf dry mass	Leaf phosphorus content	The ratio of the quantity of phosphorus of a leaf per unit dry mass.	%
Craine et al. (2009)	Nitrogen isotope ratio (d^15^N;‰)	Nitrogen isotope ratio (d^15^N;‰)	The ratio of ^15^N to^14^N of a leaf.	‰
Dawson et al. (2002)	Carbon isotope ratio (d^13^C;‰)	Carbon isotope ratio (d^13^C;‰)	The ratio of ^13^C to ^12^C of a leaf.	‰
This study		Forest-use intensity. (OG - old-growth forest, DF - degraded forest, SF - secondary forest)	A level of forest fragmentation, subjected to ongoing disturbance and/or deforestation.	
This study		DBH	Diameter at breast height	cm
